# Recent expansion of the non‐recombining sex‐linked region on *Silene latifolia* sex chromosomes

**DOI:** 10.1111/jeb.14063

**Published:** 2022-07-14

**Authors:** Dmitry A. Filatov

**Affiliations:** ^1^ Department of Plant Sciences University of Oxford Oxford UK

**Keywords:** pseudoautosomal region, pseudoautosomal boundary, recombination suppression, sex chromosome evolution, *Silene latifolia*

## Abstract

Evolution of a non‐recombining sex‐specific region on the Y (or W) chromosome (NRY) is a key step in sex chromosome evolution, but how recombination suppression evolves is not well understood. Studies in many different organisms indicated that NRY evolution often involves several expansion steps. Why such NRY expansions occur remains unclear, although it is though that they are likely driven by sexually antagonistic selection. This paper describes a recent NRY expansion due to shift of the pseudoautosomal boundary on the sex chromosomes of a dioecious plant *Silene latifolia*. The shift resulted in inclusion of at least 16 pseudoautosomal genes into the NRY. This region is pseudoautosomal in closely related *Silene dioica* and *Silene diclinis*, indicating that the NRY expansion occurred in *S. latifolia* after it speciated from the other species ~120 thousand years ago. As *S. latifolia* and *S. dioica* actively hybridise across Europe, interspecific gene flow could blur the PAR boundary in these species. The pseudoautosomal genes have significantly elevated genetic diversity (π ~ 3% at synonymous sites), which is consistent with balancing selection maintaining diversity in this region. The recent shift of the PAR boundary in *S. latifolia* offers an opportunity to study the process of on‐going NRY expansion.

## Graphical abstract

The location of the pseudoautosomal boundary in Silene latifolia and its close relatives.

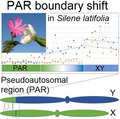

This article has a commentary by Charlesworth, https://onlinelibrary.wiley.com/doi/10.1111/jeb.14137

## 1 INTRODUCTION

Evolution of sex chromosomes is arguably one of the most significant genomic changes that leads to wide‐reaching consequences throughout the genome (Charlesworth, 2008, 2015; Mank et al., 2014; Wright et al., 2016). Despite multiple independent origins of sex chromosomes (e.g. Bachtrog et al., 2014), their properties are similar in different organisms, featuring a genetically degenerate non‐recombining sex‐specific Y (or W) chromosome (NRY) and non‐sex‐specific X (or Z) chromosome that actively recombines in homogametic sex (Charlesworth, 2008). Previous work in mammals, *Drosophila* and plants shed light on many aspects of sex chromosome evolution (Charlesworth, 2015; Wright et al., 2016). For example it is clear that sex chromosomes typically evolve from autosome(s) that acquire sex‐determining gene(s), stop recombining around that gene in the heterogametic sex (Ohno, 1967) and gradually degenerate afterwards (Charlesworth, 2008; Wright et al., 2016). However, there appear to be many ‘exceptions to the rule’ (Furman et al., 2020), such as sex chromosome turnovers that are common in some organisms (Vicoso, 2019), the extent of sex chromosome differentiaton (Darolti et al., 2019), as well as differences in pace of sex chromosome evolution between plants and animals (Bergero & Charlesworth, 2011; Chibalina & Filatov, 2011; Krasovec, Chester, et al., 2018a). It has been argued that these ‘exceptions’ reflect reasonably well understood processes (Charlesworth, 2021) and only prove the ‘rule’. Recent studies of non‐model organisms with homomorphic sex chromosomes, such as frogs (Jeffries et al., 2018; Rodrigues et al., 2017), fish (El Taher et al., 2021) and plants (Harkess et al., 2020; Muller et al., 2020) revealed a highly dynamic picture, with frequent sex chromosome turnovers (Vicoso, 2019) and occasional recombination between the X (or Z) and Y (or W) chromosomes (Rodrigues et al., 2017), which expands the ‘classic paradigm’ of sex chromosome evolution (Kratochvil et al., 2021).

Despite the progress in understanding of sex chromosome evolution, many pivotal questions remain unanswered (Furman et al., 2020; Wright et al., 2016). In particular, how nascent sex chromosomes originate in the first place, and how recombination suppression between them evolves remain poorly understood (Charlesworth, 2017, 2021; Ponnikas et al., 2018). The non‐recombining sex‐specific region tends to expand over time, including a larger proportion of the sex chromosome. Such expansions leave a characteristic signature of ‘evolutionary strata’—lower divergence between the X and Y (or Z and W) chromosomes in regions that stopped recombining more recently (‘younger strata’) compared to older non‐recombining regions (‘older strata’; Lahn & Page, 1999). Such stratification of divergence between the X and Y (or Z and W) chromosomes was reported in many organisms that evolved sex chromosomes independently from each other (e.g. Bergero et al., 2007; Lahn & Page, 1999; Zhou et al., 2014). Why such expansions of the NRY occur remains unclear and is actively discussed in the literature (Charlesworth, 2017; Jeffries et al., 2021; Lenormand & Roze, 2022; Ponnikas et al., 2018). Sexually antagonistic (SA) genes are thought to play an important role in this process (Charlesworth et al., 2014; Kirkpatrick & Guerrero, 2014; Rice, 1987), though relatively little experimental evidence in support of this plausible hypothesis is available (Charlesworth, 2018). Alternatives to the SA hypothesis, proposed to explain expansion of the NRY, include early emergence of dosage compensation (Lenormand & Roze, 2022), neutral divergence between the X and Y chromosomes (Bengtsson & Goodfellow, 1987; Ironside, 2010; Jeffries et al., 2021; Olito & Abbott, 2020) and sheltering of deleterious mutations by permanent heterozygosity in males (Charlesworth & Wall, 1999; Jay et al., 2022; Olito et al., 2022).

Evolution of a non‐recombining sex‐specific region on the Y (or W) chromosome restricts recombination in heterogametic sex to pseudoautosomal region (PAR) at the end of sex chromosomes. PAR has peculiar properties that distinguish it from both the sex chromosomes and the autosomes (Otto et al., 2011). In particular, PARs are often gene‐rich, GC‐rich and tend to have high recombination (Lien et al., 2000) and mutation (Filatov & Gerrard, 2003) rates, compared with other parts of the genome. Furthermore, due to their partial sex linkage PARs are thought to be the hotspots for the accumulation of sexually antagonistic mutations (Charlesworth et al., 2014; Kirkpatrick & Guerrero, 2014; Rice, 1987). While much research effort is focused on sex chromosomes, the PARs remain understudied, although some recent progress in understanding of PAR evolutionary dynamics has been achieved in mice (Morgan et al., 2019), humans (Ellis et al., 1990; Monteiro et al., 2021) and few plants.

In plants the analyses of PAR have, so far, been focusing on *Silene latifolia* (Campos et al., 2017; Guirao‐Rico et al., 2017; Krasovec et al., 2020; Qiu et al., 2016), although papaya (Lappin et al., 2015) and brown alga *Ectocarpus* (Avia et al., 2018; Luthringer et al., 2015) were also studied to some extent. This paper extends the analysis of the PAR in *S. latifolia* and its close relatives with the aim to better characterise the boundary between the PAR and the rest of the sex chromosome and to test whether it differs between closely related species. The shifts of the PAR boundary result in expansion of the NRY, which may occur via inversions suppressing recombination between the sex chromosomes in the heterogametic sex, as has been proposed for sex chromosomes in multiple bird lineages (Zhou et al., 2014). Alternatively, the shift of the PAR boundary may be gradual, as suggested by the reports that the PAR boundary in mice (Morgan et al., 2019) and *S. latifolia* (Krasovec et al., 2020; Qiu et al., 2016) is ‘fuzzy’.

*Silene latifolia* is a model species for studies of plant sex chromosomes (Bernasconi et al., 2009). This lineage has evolved separate sexes (dioecy) and sex chromosomes relatively recently, around 11 million years (my) ago (Krasovec, Chester, et al., 2018a). Heteromorphic X and Y chromosomes are the largest in the *S. latifolia* genome and are clearly distinguishable under the microscope (Armstrong & Filatov, 2008), which made *S. latifolia* the species of choice for the studies of plant sex chromosomes since the first half of the last century (Warmke, 1946; Westergaard, 1946, 1958). Most (>700) species in the genus *Silene* are non‐dioecious and separate sexes and sex chromosomes are clearly derived traits (Desfeux et al., 1996), making it possible to compare the sex chromosomes in the dioecious species to the ‘ancestral’ state preserved in non‐dioecious species of the same genus (Filatov, 2005).

At least one large expansion of the NRY has been reported for *S. latifolia* sex chromosomes, which resulted in formation of two evolutionary strata approximately 11 and 6 million years ago (Bergero et al., 2007; Filatov, 2005; Krasovec, Chester, et al., 2018a). A recent study (Campos et al., 2017) making a comparison of sex chromosomes between closely related *S. latifolia* and *S. dioica* that share the same or very similar sex chromosomes (Filatov et al., 2009), suggested a second NRY expansion that occurred in *S. latifolia* since its divergence from *S. dioica* only ~120 thousand years (Liu et al., 2020). Unfortunately, the previous analysis of the PAR boundary shift had limited resolution as it was based on six genes, only two of which were located in the region that is pseudoautosomal in *S. dioica* and sex‐linked in *S. latifolia*. This paper extends that analysis to a larger set of genes near the PAR boundary to re‐evaluate the evidence for the recent NRY expansion in *S. latifolia*, to establish the boundaries and to study the possible causes of NRY expansion. The analyses reported below confirm the recent expansion of the NRY in *S. latifolia* and reveal that the newly added evolutionary stratum contains at least 16 genes, but it is much smaller than the older strata on *S. latifolia* sex chromosomes that contain hundreds of genes (Bergero et al., 2015; Bergero & Charlesworth, 2011; Chibalina & Filatov, 2011; Papadopulos et al., 2015). This illustrates that recombination suppression can progress gradually or in small steps that shrink the PAR and result in NRY expansion.

## 2 MATERIALS AND METHODS

### 2.1 Plant material

This work is based on plants from *S. latifolia* genetic cross df108 described previously (Papadopulos et al., 2015). The df108 family is an F2 generation from crossing of maternal (fSa985) and paternal (Sa984) plants, with transcriptomes sequenced for the parents and 52 F2 progeny (see Table S1). Furthermore, the work involved wild accessions from three dioecious Silene species (*S. latifolia*, *S. dioica* and *S. diclinis*) and a non‐dioecious *Silene vulgaris* that was used as an outgroup (Table S1). The plants were grown in the glasshouse (20°C and 15 h lighting) from seed collected between 2000 and 2006 all over Europe.

### 2.2 Sequence data

Transcriptome sequence data for the genetic cross df108 were generated in the previous study (Papadopulos et al., 2015) and is available from NCBI bioproject PRJNA289919. RNA‐seq data for wild samples included both published and unpublished datasets (Table S1). RNA was extracted from actively growing shoots and flower buds using a Qiagen RNeasy Plant Mini Kit, including the optional on‐column DNAse digestion. Isolation of mRNA, cDNA synthesis and high‐throughput sequencing were conducted according to the standard Illumina RNA‐Seq procedure at the Oxford genomics facility of the Wellcome Trust Centre for Human Genetics (WTCHG). All newly generated sequence data were submitted to the NCBI SRA database under bioproject number PRJNA826722.

Raw transcriptome sequence data were trimmed with trimmomatic v.0.30 (Bolger et al., 2014) with default parameters and mapped to the reference female transcriptome (Chibalina & Filatov, 2011; Papadopulos et al., 2015) with Bowtie2 (Langmead & Salzberg, 2012). The numbers of reads that successfully mapped to reference for each transcriptome are listed in Table S1. Single nucleotide polymorphisms (SNPs) were called with GATK HaplotypeCaller (McKenna et al.,  2010) and the resulting multisample vcf file was imported to proseq3 v3.997 (Filatov, 2009). The dataset was filtered with proseq3 to leave only the genes which are located close to the PAR boundary in the previously published genetic map (Papadopulos et al., 2015). proseq3 was also used to assign coding regions to all sequences, based on annotation in the reference transcriptome (Chibalina & Filatov, 2011; Papadopulos et al., 2015) and filter sites in the alignments to reduce the data to 4‐fold degenerate sites. Nucleotide diversity (π eq. 10.5 in (Nei, 1987)), *K*_st_* (Hudson et al., 1992), Tajima's *D* (Tajima, 1989) and Kelly's ZnS (Kelly, 1997) values were calculated with proseq3. HKA test (Hudson et al., 1987) was done using the ‘direct mode’ in dnasp v6.12.03 (Rozas et al., 2017), while MLHKA test was done with mlhka program (Wright & Charlesworth, 2004) that was integrated in proseq3. The 30 autosomal genes used as the reference set in the mlhka analysis were randomly selected from the genes mapped previously (Papadopulos et al., 2015). The sequence divergence from the outgroup *S. vulgaris* was calculated with mega v10.1.5 (Kumar et al., 2018).

### 2.3 Identification of the putative Y‐linked alleles

**FIGURE 1:**
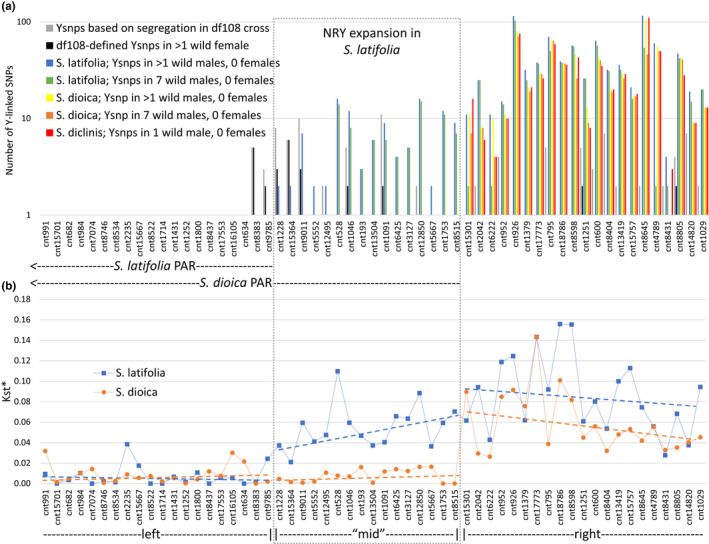
Location of the PAR boundary in *Silene latifolia* and its relatives. PAR extends to the left of the plot and the differentiated part of the sex chromosomes extends to the right. (a) The number of putative Y‐linked SNPs per gene identified as segregating from father to sons in family df108 (Papadopulos et al., 2015) (grey bars) and as male‐specific SNPs in the wild accessions of *S. latifolia*, *S. dioica* and *S. diclinis* (blue + green, yellow + orange and red bars, respectively). The blue + green bars for *S. latifolia* and yellow + orange bars for *S. dioica* show the numbers of Y‐SNPs present in at least two and in all seven wild males of each species analysed, respectively. The black bars show the numbers of putative ‘Y‐SNPs’ (defined by segregation in df108 cross) present in wild *S. latifolia* females. (b) *K*_st_* (Hudson et al., 1992) between males and females in the genes adjacent to the PAR boundary in *S. latifolia* (blue squares) and *S. dioica* (orange dots). *K*_st_* for *S. diclinis* was not calculated because only two accessions were available for this species. Dashed lines show linear regression separately fitted to *S. latifolia* and *S. dioica* data in the left, middle and right parts of the plot

Transcriptome sequence data from parents (fSa985 and Sa984; Table S1) and 52 progeny (20 males and 32 females) of *S. latifolia* genetic cross df108 (Papadopulos et al., 2015) were used to identify the putative Y‐linked alleles. The Y‐alleles (‘Y‐SNPs’) were identified as alleles always inherited from father to male progeny and never to female progeny across two generations. The corresponding X‐alleles at these loci are always inherited from father to female progeny. The presence of these Y‐SNPs was further analysed in wild male and female plants in three dioecious Silene species. To allow for missing data, two stringency thresholds were used, requiring the presence of the putative Y‐SNP in (i) at least two wild males and no females and (ii) in all seven wild males and none of the females. This yielded sets of SNPs that are Y‐linked in both the genetic cross and the wild samples, as well as the SNPs that show Y‐linked segregation pattern, but are male‐specific in one or more species, as shown on Figure 1a.

### 2.4 Measuring divergence between homologous X‐ and Y‐linked genes

Sequence reconstruction for the Y‐linked homologs of X‐linked genes was done as described previously (Chibalina & Filatov, 2011, Papadopulos et al., 2015). Briefly, to reconstruct the sequence of the Y‐linked genes, Y‐SNPs were used to identify the sequence reads (along with their paired reads) corresponding to the Y‐linked allele and to call Y‐consensus with proseq3 (Filatov, 2009). The homologous X‐ and Y‐linked sequences were aligned with muscle (Edgar, 2004). The resulting alignments were used to calculate sequence divergence at silent sites with proseq3 (Filatov, 2009).

### 2.5 Gene expression analysis

Gene expression was measured as the number of read fragments per million reads mapped per kilobase of reference sequence (FPKM) calculated with RSEM (Li & Dewey, 2011). FPKM was calculated for each gene of each individual and gene expression in males and females was compared as log2(median(femaleFPKM)) − log2(median(maleFPKM)).

## 3 RESULTS

### 3.1 The data

The analysis presented below included previously published transcriptome sequence data from 52 progeny of *S. latifolia* genetic cross df108 (Papadopulos et al., 2015) as well as newly sequenced and previously published data for 14 *S. latifolia* and 14 *S. dioica* wild accessions grown from seed collected across Europe (Table S1). Each accession came from a different location to avoid the effects of population structure at the local scale (Wakeley, 1998). These samples include both males and females, which are analysed jointly or separately, depending on the type of analysis. Furthermore, the dataset included a male and a female *Silene diclinis* plants grown from seed collected in Valencia (Spain), as well as a non‐dioecious *Silene vulgaris* that was used as an outgroup. As the aim of this work is to analyse and compare the PAR boundary region in *S. latifolia* and its close relatives, the analyses below focus on 57 genes (Table S2) in the proximity of the PAR boundary (according to the genetic map (Papadopulos et al., 2015)) for which sufficient sequence data was available for both sexes in *S. latifolia*, *S. dioica* and *S. diclinis* (Table S1).

### 3.2 The location of the PAR boundary in *S. latifolia* and *S. dioica*

The location of the PAR boundary was established using a combination of segregation analysis in a previously published genetic cross df108 (Papadopulos et al., 2015) and the analysis of polymorphism in wild accessions of *S. latifolia*, *S. dioica* and *S. diclinis*. In the genetic cross, the putatively Y‐linked SNPs were identified as SNPs inherited from father to sons and not daughters. Gene cnt8383 and all 38 genes located proximally (to the right in Figure 1a) contain SNPs segregating from father to sons and not to daughters, indicating some degree of sex linkage. No such putatively Y‐linked SNP were detected in the 18 genes located distally (to the left in Figure 1a) to the gene cnt8383 despite multiple SNPs segregating at these genes, suggesting their pseudoautosomal location.

Segregation in a genetic cross cannot detect rare recombination events and the size of fully sex‐linked regions may be overestimated (e.g. (Charlesworth, 2021; Krasovec et al., 2020)). Thus, the presence of male‐specific SNPs in unrelated individuals of the same species is a more accurate indicator of gene sex linkage. To account for inevitable missing data, two stringency levels were used, requiring male‐specific SNPs to be found in at least two and in all seven analysed wild *S. latifolia* males, respectively (Figure 1a). In both cases, the SNPs present in wild females were excluded. No male‐specific SNPs were identified in wild *S. latifolia* samples in cnt8383 and in its proximal neighbour gene cnt9785, but nearly all the genes located more proximally contained at least one male‐specific SNP, indicating complete or nearly complete sex linkage of the genes located proximally (to the right in Figure 1a) to cnt9785. Using the stricter requirement of Y‐SNP presence in all seven wild *S. latifolia* males (green bars in Figure 1a), the PAR boundary may be located a bit more proximally—around the gene cnt5552. This is also consistent with the presence of segregation‐defined Y‐SNPs in wild females in the genes located distally to cnt5552 (black bars in Figure 1a). The few cases of Y‐SNP presence in females for genes located proximally to cnt5552 may be due to occasional SNP mis‐calling or genuine rare recombination events between the X and Y chromosomes near the PAR boundary. Consistent with the location of the PAR boundary in this region, *K*_st_* (an *F*_st_‐like statistic for sequence data (Hudson et al., 1992)) between *S. latifolia* males and females is nearly zero (average *K*_st_* = 0.007) in the genes located distally to cnt9785. All more proximal genes have much higher *K*_st_* values (average *K*_st_* = 0.072; Figure 1b) that are significantly different from zero (permutation test, *p* < 0.0001).

The same analysis in *S. dioica* revealed male‐specific SNPs in cnt15301 and the proximal genes, indicating sex linkage of that region. None of the genes located distally to cnt15301 contain any male‐specific SNPs (Figure 1a), indicating that this region is pseudoautosomal in *S. dioica*. Furthermore, *K*_st_* between *S. dioica* males and females is low (average *K*_st_* = 0.008) in all the genes distal to cnt15301, while *K*_st_* is higher for that gene and all proximally located genes (average *K*_st_* = 0.071; Figure 1b). These data are consistent with the location of the *S. dioica* PAR boundary between the genes cnt8515 and cnt15301 (Figure 1). While too few samples are available for *S. diclinis* to test for sex linkage of individual SNPs, it is still possible to check the presence of putative Y‐SNPs in the male and the female of that species. The SNPs identified as Y‐linked in *S. latifolia* and *S. dioica* (as described above), are often present in the *S. diclinis* female for the genes located distally to cnt15301, while this is never the case for proximally located genes, where the putatively Y‐linked SNPs are present in the male, but not in the female (Figure 1a). This is consistent with the same location of the PAR boundary in *S. dioica* and *S. diclinis*. For convenience and brevity, the three parts of the analysed region will be referred to as the ‘left’, the ‘middle’ (or ‘mid’) and the ‘right’ (Figure 1).

### 3.3 Genetic diversity near the PAR boundary

**FIGURE 2:**
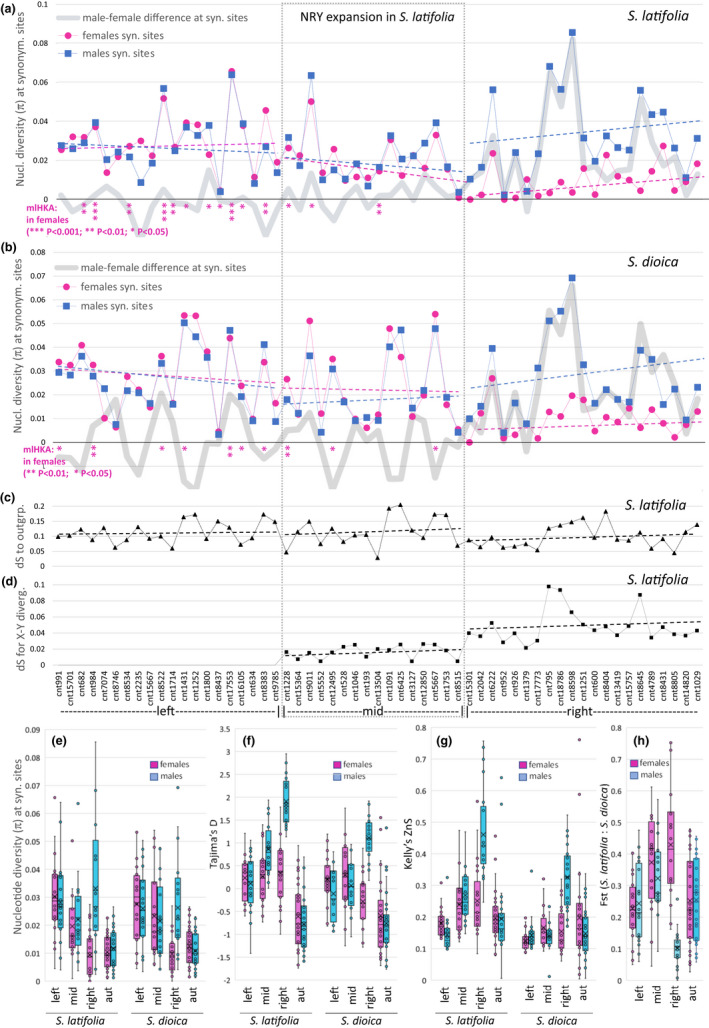
Level and patterns of DNA sequence polymorphism (a, b, e‐h) in *Silene latifolia* and *S. dioica* and silent divergence from the outgroup *S. vulgaris* (c) and between X‐ and Y‐linked gametologs (d) in the genes near the PAR boundary. The order of genes in the panels (a–d) is the same and the gene names are shown below panel (d). Dashed lines in panels (a–d) show linear regression separately fitted to data in the left, middle and right parts of the plot. The wide grey lines in panels (a) and (b) show the difference between sexes in nucleotide diversity per site (π) and the red stars below the zero line show significance (***p < 0.001; **p < 0.01; *p < 0.05 [with starts shown vertically]) for individual genes in mlhka test (Wright & Charlesworth, 2004) with the 21 X‐linked genes from the right part used as a reference set. For autosomal genes used as a reference see Table S2. (d) d*S* between X and Y is shown only for genes in the mid and right regions; (e–h) Box‐and‐whiskers plots showing the quartiles, the mean (the cross) and the actual data points (circles) for the distributions of summary statistics in the genes in the left, the middle and the right parts of the region, as well as in 30 autosomal genes (aut; Table S4) randomly chosen from the previously mapped genes (Papadopulos et al., 2015), for females (red) and males (blue) in *S. latifolia* and *S. dioica*. (a), (b), (e) Nucleotide diversity per site (π (Nei, 1987)) at 4‐fold degenerate codon positions; (f) Tajima's *D* (Tajima, 1989), (g) Kelly's *Z*_nS_ (Kelly, 1997) and (h) *F*_st_ between *S. latifolia* and *S. dioica*

The genetic diversity was measured for synonymous (four‐fold degenerate) sites (Figure 2a,b,e; Table S2) to minimize the effect of purifying selection on the level of diversity at non‐silent sites. Overall, genetic diversity is similar in the two species (Figure 2e), and the difference between *S. latifolia* and *S. dioica* females is not significant (π_dioF_ = 0.0199 and π_latF_ = 0.0196; paired *t*‐test, *p* = 0.837). However, the diversity in *S. dioica* males is slightly but significantly lower compared to *S. latifolia* males (π_dioM_ = 0.024 and π_latM_ = 0.028; paired *t*‐test, *p* = 0.021). In both species the overall diversity in males is significantly higher than in females (paired *t*‐tests, *p* < 0.01), which is driven primarily by much higher polymorphism in the males compared to females in the right part of the region that is sex‐linked in both species (π_latMright_ = 0.0332, π_latFright_ = 0.0093, *p* < 0.0001; π_dioMright_ = 0.0263, π_dioFright_ = 0.0097, *p* < 0.0001). The differences in genetic diversity between the sexes are not significant in the left (π_latMleft_ = 0.0281, π_latFleft_ = 0.0303, *p* = 0.22; π_dioMleft_ = 0.0260, π_dioFleft_ = 0.0276, *p* = 0.184) or the middle (π_latMmid_ = 0.0220, π_latFmid_ = 0.0196, *p* = 0.22; π_dioMmid_ = 0.0215, π_dioFmid_ = 0.0233, *p* = 0.184) parts of the region. The higher diversity in males in the right part of the region is likely driven by divergence between the X‐ and the Y‐linked alleles, while recombination in the PAR in males prevents such divergence (Figure 2d). This divergence is significantly higher in the right compared to the middle region (mean d*S*_XYright_ = 0.049; d*S*_XYmid_ = 0.016; *t*‐test, *p* < 10^−6^), consistent with very recent recombination suppression in the mid region in *S. latifolia* males.

Synonymous genetic diversity is consistently high in the pseudoautosomal genes of both species (π_latMleft_ = 0.0281; π_latFleft_ = 0.0303; π_dioMleft_ = 0.0260; π_dioFleft_ = 0.0276). Focusing on the females to exclude the contribution of Y‐linked alleles, these diversity values are significantly (*t*‐tests, *p* < 0.001) higher than the synonymous diversity in the X‐linked genes in the right part of the region (π_latFright_ = 0.0093; π_dioFright_ = 0.0097). Furthermore, synonymous diversity in the PAR (left region) is significantly (*t*‐test, *p* < 0.003) higher compared with autosomal genes (Figure 2e; Table S4) for both males (π_latMaut_ = 0.0154; π_dioMaut_ = 0.0146) and females (π_latFaut_ = 0.0143; π_dioFaut_ = 0.0165). The elevated polymorphism in the pseudoautosomal genes could be caused by higher mutation rate in this region, as was reported for the PAR in primates (Filatov & Gerrard, 2003). If this were the case, the pseudoautosomal genes would be expected to have higher interspecific divergence compared to genes elsewhere in the genome. However, synonymous divergence from the homologs in the outgroup *S. vulgaris* is similar in the left, middle and right parts of the region (Figure 2c) and the difference between them is not significant (*t*‐test, *p* > 0.2). Furthermore, the analysis of synonymous diversity in *S. latifolia* females, taking divergence from the outgroup *S. vulgaris* into account with HKA test (Hudson et al., 1987), revealed significantly (χ^2^ = 4.962, *p* = 0.0259) higher female diversity in the concatenated pseudoautosomal compared with the concatenated X‐linked genes (the left and the right parts of the region in Figure 2a–c, respectively). Similar comparison of the left region with the previously mapped (Papadopulos et al., 2015) autosomal genes (Table S4) is also significant (χ^2^ = 5.104, *p* = 0.0239). The same HKA test for the comparison of synonymous variation in the left and the right regions in *S. dioica* females is not significant (χ^2^ = 2.383, *p* = 0.1226), but the comparison of the left region with autosomal genes is significant (χ^2^ = 4.368, *p* = 0.0366). As HKA test uses divergence from the outgroup to adjust for possible mutation rate differences between the genes, higher mutation rate in the PAR cannot explain the excess of genetic diversity in the pseudoautosomal genes compared with the X‐linked genes in *S. latifolia*.

Synonymous genetic diversity at individual genes in the left and middle parts of the region was compared to the X‐linked genes in the right part and to autosomal genes using mlhka (Wright & Charlesworth, 2004), a maximum likelihood implementation of the HKA test (Hudson et al., 1987). In *S. latifolia* females, this analysis revealed significant excess of genetic diversity in 10 and 8 out of 20 pseudoautosomal genes (left region) when compared to the X‐linked (right region) and autosomal genes, respectively (Figure 2a; Table S2). The same analysis in *S. dioica* identified 7 and 6 PAR genes in the left region, compared with the X‐linked and autosomal genes, respectively (Figure 2b; Table S2). One of the *S. latifolia* PAR genes, cnt8437, contained significantly less genetic diversity compared with that in the X‐linked and autosomal genes (Figure 2a; Table S2). In both species only three out of 16 ‘mid’ genes contained significant excess of polymorphism compared with the X‐linked and autosomal genes (Figure 2a; Table S2). None of the X‐linked genes (right region) showed significant excess of diversity compared with autosomal genes in both species, but three and one of the X‐linked genes showed significant lack of diversity in *S. latifolia* and *S. dioica*, respectively (Table S2).

As *S. latifolia* and *S. dioica* often hybridise in the wild, differential interspecific gene flow in different genomic regions could, at least partly, account for elevated genetic diversity in the PAR. Indeed, *F*_st_ between the species (Figure 2h) is significantly lower in the PAR compared to the mid and right regions in females (*t*‐test, *p* < 0.0001) and in the mid region in males (*t*‐test, *p* < 0.05), consistent with reduced interspecific gene flow on the *S. latifolia* and *S. dioica* X chromosomes, as reported in the previous study (Hu & Filatov, 2016). However, *F*_st_ in the PAR is similar (*t*‐test, *p* > 0.05) to that for the autosomal genes (Figure 2h), indicating that differential interspecific gene flow is unlikely to be the cause of high PAR genetic diversity. This is consistent with the conclusion of the previous study (Guirao‐Rico et al., 2017) that explicitly modelled the effect of interspecific gene flow between *S. latifolia* and *S. dioica* on patterns of diversity in the PAR. It is worth noting that reduced *F*_st_ in the right region for males is caused by sharing of numerous Y‐SNPs by the two species because the divergence of the X‐ and Y‐linked gametologs for the genes in the right region predates speciation of *S. latifolia* and *S. dioica*.

### 3.4 Patterns of genetic diversity around the PAR boundary

The overall frequency spectrum of polymorphisms, summarised by the Tajima's *D* statistic (Tajima, 1989) is consistent with the left part of the region being pseudoautosomal in both species, the right being sex‐linked and the middle being sex‐linked in *S. latifolia* and pseudoautosomal in *S. dioica* (Figure 2f; Table S3). Although Tajima's D varies among the genes, the overall distributions for the females in the left, middle and right parts of the region are quite similar and close to zero. The same is the case for males in the left (pseudoautosomal) part for both species and for *S. dioica* males in the middle part (Figure 2f). The males in the right (sex‐linked) part of the region show strongly positive Tajima's *D*, as would be expected for the region where Y‐linked SNPs contribute an excess of intermediate frequency polymorphisms. This is less pronounced but still detectable in the sex‐linked middle part of the region for *S. latifolia* males, but not in *S. dioica*, where this part is likely pseudoautosomal (Figure 2f).

The patterns of linkage disequilibrium (LD) within genes, summarised by the Kelly's *Z*_nS_ statistic (Kelly, 1997) are also consistent with sex linkage of the right part of the region in both species and the middle part in *S. latifolia*, but not in *S. dioica* (Figure 2g; Table S3). Sex linkage is expected to inflate LD in the heterogametic sex and this is clearly visible in the *Z*_nS_ distribution for the right region in males of both species (Figure 2g). LD is the lowest in the left region of both species, as expected for the genes in the PAR that actively recombine in both males and females. The distribution of *Z*_nS_ in the middle region in *S. dioica* is similar to that in the left region in both sexes, which is consistent with the middle region being pseudoautosomal in this species. On the other hand, *Z*_nS_ in the middle region in *S. latifolia* is intermediate between the left and the right regions, consistent with recent sex linkage of the middle region in *S. latifolia*.

### 3.5 Female and male expression of the genes around the PAR boundary

**FIGURE 3:**
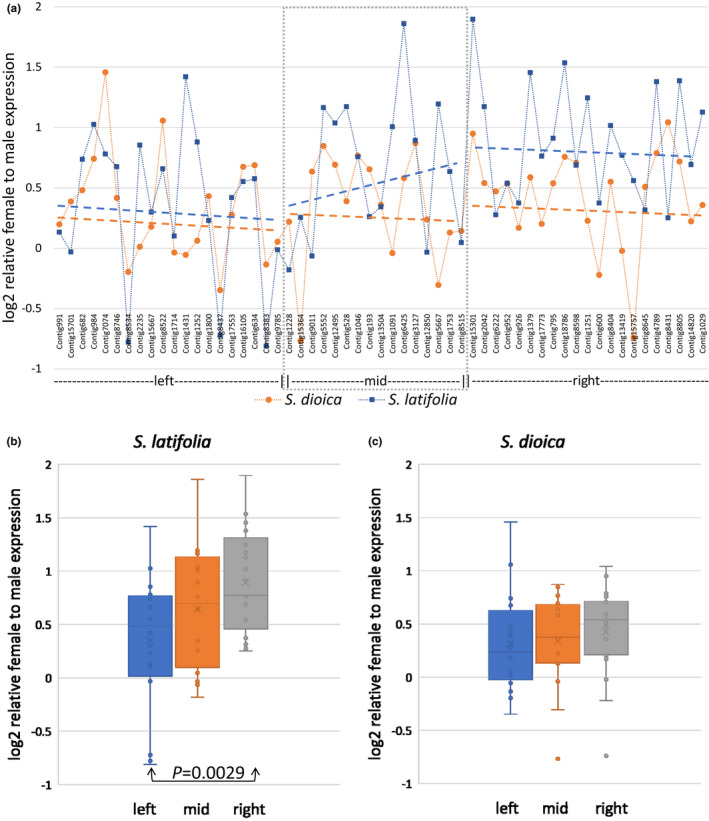
Relative expression of genes in females and males. The Y‐axis shows the log2(median(femaleFPKM)) − log2(median(maleFPKM)). The only significant comparison (*t*‐test; *p* = 0.0029) was between the left and the right regions in *Silene latifolia*; shown with arrows. Dashed lines in the panel A show linear regression separately fitted to data in the left, middle and right parts of the plot

Most *S. latifolia* and *S. dioica* genes near the PAR boundary show female expression bias (Figure 3a). The strongest bias was detected in the ‘right’ (sex‐linked) region in *S. latifolia* (Figure 3b), which may reflect degeneration of Y‐linked alleles, resulting in nearly two‐fold excess of expression in females. This bias in the right region is significantly (*t*‐test; *p* = 0.0029) stronger compared with the left region, with the mid region showing intermediate level of sex‐bias. No significant difference in female bias was detected between the three regions in *S. dioica* (Figure 3c).

## 4 DISCUSSION

The results presented above are consistent with the recent expansion of the NRY region in *S. latifolia* since the divergence from its close relatives *S. dioica* and *S. diclinis*. In particular, the presence of male‐specific SNPs and elevated *K*_st_* between sexes in the middle part of the region in *S. latifolia* but not in *S. dioica* or *S. diclinis* (Figure 1) are consistent with this region being sex‐linked in *S. latifolia*, and pseudoautosomal in *S. dioica* and *S. diclinis*. However, alternative explanations for these observations are also possible. For example, a translocation of autosomal genes to the Y chromosome in *S. latifolia* would result in the presence of male‐specific SNPs for the translocated genes. However, this would imply autosomal location of the genes in the middle region (+ the copy translocated to the Y chromosome), while the genetic mapping demonstrated the location of these genes on the X chromosome near the PAR boundary (Papadopulos et al., 2015). Another possibility is that the middle region may be fully sex‐linked in all the species analysed and the lack of male‐specific SNPs in *S. dioica* and *S. diclinis* may be caused by a deletion of Y‐linked alleles in these species. However, this would either imply independent deletions of the same region in *S. dioica* and *S. diclinis*, which is unlikely, or it implies that Y chromosomes of *S. dioica* and *S. diclinis* are more closely related to each other than to *S. latifolia*, which contradicts the available evidence. *S. latifolia* and *S. dioica* sex chromosomes share the same structure (Filatov et al., 2009), which differs from that in *S. diclinis* (Howell et al., 2009). Furthermore, *S. latifolia* and *S. dioica* are cross‐compatible and widely hybridise in the wild, while *S. diclinis* is partially reproductively isolated, suggesting that it is more diverged from the other two species. This is also consistent with sequence divergence indicating that *S. diclinis* is slightly more diverged compared with *S. latifolia* and *S. dioica* (Krasovec, Nevado, & Filatov, 2018b). Thus, a shift in the PAR boundary in *S. latifolia* appears to be a more likely explanation to the results presented above. Lower synonymous divergence between the X‐ and Y‐linked gametologs in the mid compared with the right region (Figure 2d) is also consistent with the recent shift of the PAR boundary in *S. latifolia*.

How big is the region added to the *S. latifolia* NRY as a result of recent PAR boundary shift? Our analysis identified 16 expressed genes that are located in the region of recent NRY expansion in *S. latifolia* (Figure 1). While it is difficult to assess the physical size of that region without high quality genome sequence, it is possible to evaluate its size given the number of genes in that region and the average gene density for genomes of similar size, such as the tobacco (*Nicotiana tabacum*) genome (Sierro et al., 2014). The gene density in that genome is roughly 15 to 20 genes per megabase, which suggests that the size of the recent NRY expansion in *S. latifolia* is at least 1 Mb long. This is a very crude estimate as gene density can vary by an order of magnitude between actively and rarely recombining regions of the genome. Also, it is likely a minimal size estimate because only a subset of actively expressed genes have been identified in this region. Although small, it is comparable to or larger than the sizes of the entire NRY in many of the plant species analysed, such as persimmon (NRY length ~1 Mb; (Akagi et al., 2014)), kiwi fruit (NRY length <1 Mb; (Akagi et al., 2019)) and Asparagus (NRY length <1 Mb (Harkess et al., 2017)). Thus, the recent NRY expansion in *S. latifolia* is substantial in size, though it is clearly much smaller than the older ‘evolutionary strata’ in *S. latifolia* that include hundreds of genes and are likely hundreds of megabases in size. It remains unclear whether the older larger strata originated via instantaneous step‐wise recombination cessation in the entire stratum, or the process was more gradual. If recombination cessation occurred in multiple small steps, the recent expansion of the NRY in *S. latifolia* described above may represent one of such steps and it could be used to study the process of on‐going NRY expansion.

The mechanisms involved in recombination suppression on the sex chromosomes are not well understood, but it is often assumed that inversions play a major role in recombination suppression and NRY expansion on sex chromosomes (Charlesworth, 2017). The spread of inversions expanding the non‐recombining region may be driven by the advantage conferred by sheltering deleterious mutations by permanent heterozygosity in males (Charlesworth & Wall, 1999; Jay et al., 2022; Olito et al., 2022). It was recently shown that such inversions have a limited window of opportunity to fix (while still lightly loaded by mutations) and the outcome of this process is dependent on inversion size, with small inversions having elevated probability of fixation compared to neutrality (Olito et al., 2022). The spread of such an inversion by this or some other selective mechanism should result in loss of polymorphism in the inverted region, providing an explanation for lower genetic diversity in the middle and the right regions in *S. latifolia* (Figure 2a,e). However, this mechanism does not provide an explanation for the difference in diversity between the PAR and differentiated part (left and right regions in Figure 2b,e) in *S. dioica*, where no recent shifts of the PAR boundary were detected.

An inversion is expected to suppress recombination at the same time all over the inverted region. It is curious that *K*_st_* in *S. latifolia* increases with distance from the PAR (in the middle part in Figure 1b), which is not expected if recombination cessation occurred simultaneously for all the genes in the region, for example due to an inversion. The gradient in *K*_st_* could be created by a gradient in the extent of sex linkage along this region or by a gradual rather than stepwise shift of the NRY boundary. Gradual recombination suppression was hypothesized to be caused by neutral divergence between the PAR genes closely linked to the X and Y chromosomes (Bengtsson & Goodfellow, 1987; Ironside, 2010; Jeffries et al., 2021; Olito & Abbott, 2020). This hypothesis predicts elevated genetic diversity (due to X:Y divergence) only in the PAR region immediately adjacent to the PAR boundary, while the analyses presented above do not reveal such localised effect in proximal PAR genes (Figure 2a,b). It is possible that the PAR region analysed is too small to detect the expected pattern of diversity reduction in the PAR with distance from the PAR boundary. However, given the length of the analysed PAR region in the genetic map is ~7 cM (Table S2), the distal genes in this region should be sufficiently decoupled from the differentiated part of sex chromosomes to reveal lower diversity compared to the proximal PAR genes.

Selection to link sexually antagonistic (SA) genes to sex is thought to be a plausible cause for the expansion of sex‐linked regions by inclusion of ever larger proportion of pseudoautosomal genes in the NRY (Rice, 1987). SA selection in the PAR is expected to create distinctive footprints in DNA sequence variation, such as local divergence between the Y‐ and the X‐linked alleles or between males and females, which can be detected as peaks of *F*_st_ (or *K*_st_*) between the sexes and elevated local genetic diversity (Kirkpatrick & Guerrero, 2014; Qiu et al., 2013). The analyses presented above reveal significantly elevated genetic diversity in the PAR genes, compared with the X‐linked genes, adjusting for ploidy and possible mutation rate difference in the HKA (Hudson et al., 1987) and mlhka (Wright & Charlesworth, 2004) tests. Balancing selection due to partial sex linkage in the PAR can only elevate diversity in the gene(s) immediately adjacent to the PAR boundary (Kirkpatrick et al., 2010). Qiu et al. (2016) estimate that in *S. latifolia* the region of elevated divergence due to sex linkage can be as short as half a kilobase (Qiu et al., 2016). Thus, balancing selection due to partial sex linkage is likely insufficient to elevate diversity in multiple pseudoautosomal genes, as shown on Figure 2a,b. While balancing selection due to sex linkage can only elevate diversity in the gene(s) immediately adjacent to the PAR boundary, SA selection in pseudoautosomal gene(s) could extend this effect to a much wider region in the PAR (Kirkpatrick & Guerrero, 2014). Thus, the presence of elevated polymorphism in multiple PAR genes is consistent with SA at some of the genes in the PAR.

Can interspecific gene flow blur the PAR boundary in *S. latifolia* and *S. dioica*? *S. latifolia* and *S. dioica* diverged about 120 thousand years ago and they widely hybridise across Europe (Liu et al., 2020; Muir et al., 2012). If these species differ in the location of the PAR boundary, their on‐going hybridisation may blur the location of the PAR boundary, which could, at least partly, account for the ‘fuzziness’ of the PAR boundary previously reported in *S. latifolia* (Krasovec et al., 2020; Qiu et al., 2016). However, the effect of interspecific hybridisation on the location of the PAR boundary may depend on whether the X or the Y chromosome play the primary role in recombination suppression in *S. latifolia* middle region. Interspecific introgression of the Y chromosome is likely to be a rare event because *S. latifolia* Y chromosome includes hundreds, if not thousands, of genes completely linked together, so its introgression between the species may be more detrimental compared to introgression of individual genes on recombining chromosomes. Thus, if recombination suppression in the middle region primarily depends on the Y chromosome (e.g. because suppression is caused by an inversion on *S. latifolia* Y, including part of the PAR adjacent to the boundary), then interspecific gene flow for the X‐linked genes that occurs more actively than for the Y chromosome, may not affect the location of the PAR boundary. However, if recombination suppression in the *S. latifolia* middle region depends on the X, or on both X and Y chromosomes, then gene flow between *S. latifolia* and *S. dioica* may blur the location of the PAR boundary in either species. This suggests that interspecific gene flow may play a role in evolution of NRY size.

## 5 CONCLUSIONS

Evolution of the non‐recombining region is the central process in sex chromosome evolution, yet relatively little is known about the mechanisms involved in this process (Bergero & Charlesworth, 2009; Charlesworth, 2017). The region adjacent to the PAR boundary may provide the clues of how cessation of recombination evolves and how NRY expands. This study extends the work devoted to the location of the PAR boundary and analysis of the adjacent region in *S. latifolia* and closely related species (Bergero et al., 2013; Campos et al., 2017; Guirao‐Rico et al., 2017; Krasovec et al., 2020; Qiu et al., 2016). The analyses presented above indicate that the PAR boundary shift has occurred in *S. latifolia* since its divergence from its close relatives *S. dioica* and *S. diclinis*. The region newly added to the non‐recombining region in *S. latifolia* is smaller than the two older evolutionary strata described in this species, but it contains at least 16 expressed genes and is likely to be over a megabase long. Significantly elevated diversity at the pseudoautosomal genes are consistent with balancing and SA selection maintaining polymorphism in the PAR, which is in line with theoretical expectations for partially sex‐linked genes near the PAR boundary (Kirkpatrick et al., 2010; Kirkpatrick & Guerrero, 2014).

## AUTHOR CONTRIBUTIONS

DAF designed the study, generated the data, conducted the analyses and wrote the paper.

## CONFLICT OF INTEREST

The author has no conflict of interest to declare.

### PEER REVIEW

The peer review history for this article is available at https://publons.com/publon/10.1111/jeb.14063.

## Supporting information


Figure S1

Tables S1‐S4

## Data Availability

The data that support the findings of this study are openly available in NCBI database at https://www.ncbi.nlm.nih.gov/, all reference numbers are listed in Table S1.

## References

[jeb14063-bib-0001] Akagi, T. , Henry, I. M. , Tao, R. , & Comai, L. (2014). A Y‐chromosome‐encoded small RNA acts as a sex determinant in persimmons. Science, 346, 646–650. 25359977 10.1126/science.1257225

[jeb14063-bib-0002] Akagi, T. , Pilkington, S. M. , Varkonyi‐Gasic, E. , Henry, I. M. , Sugano, S. S. , Sonoda, M. , Firl, A. , McNeilage, M. A. , Douglas, M. J. , Wang, T. , Rebstock, R. , Voogd, C. , Datson, P. , Allan, A. C. , Beppu, K. , Kataoka, I. , & Tao, R. (2019). Two Y‐chromosome‐encoded genes determine sex in kiwifruit. Nature Plants, 5, 801–809. 31383971 10.1038/s41477-019-0489-6

[jeb14063-bib-0003] Armstrong, S. J. , & Filatov, D. A. (2008). A cytogenetic view of sex chromosome evolution in plants. Cytogenetic and Genome Research, 120, 241–246. 18504353 10.1159/000121073

[jeb14063-bib-0004] Avia, K. , Lipinska, A. P. , Mignerot, L. , Montecinos, A. E. , Jamy, M. , Ahmed, S. , Valero, M. , Peters, A. F. , Cock, J. M. , Roze, D. , & Coelho, S. M. (2018). Genetic diversity in the UV sex chromosomes of the brown alga *Ectocarpus* . Genes, 9, 286. 29882839 10.3390/genes9060286 PMC6027523

[jeb14063-bib-0005] Bachtrog, D. , Mank, J. E. , Peichel, C. L. , Kirkpatrick, M. , Otto, S. P. , Ashman, T. L. , Hahn, M. W. , Kitano, J. , Mayrose, I. , Ming, R. , Perrin, N. , Ross, L. , Valenzuela, N. , Vamosi, J. C. , & The Tree of Sex Consortium . (2014). Sex determination: Why so many ways of doing it? PLoS Biology, 12, e1001899. 24983465 10.1371/journal.pbio.1001899 PMC4077654

[jeb14063-bib-0006] Bengtsson, B. O. , & Goodfellow, P. N. (1987). The effect of recombination between the X and Y chromosomes of mammals. Annals of Human Genetics, 51, 57–64. 3674748 10.1111/j.1469-1809.1987.tb00865.x

[jeb14063-bib-0007] Bergero, R. , & Charlesworth, D. (2009). The evolution of restricted recombination in sex chromosomes. Trends in Ecology & Evolution, 24, 94–102. 19100654 10.1016/j.tree.2008.09.010

[jeb14063-bib-0008] Bergero, R. , & Charlesworth, D. (2011). Preservation of the Y transcriptome in a 10‐million‐year‐old plant sex chromosome system. Current Biology, 21, 1470–1474. 21889891 10.1016/j.cub.2011.07.032

[jeb14063-bib-0009] Bergero, R. , Forrest, A. , Kamau, E. , & Charlesworth, D. (2007). Evolutionary strata on the X chromosomes of the dioecious plant *Silene latifolia*: Evidence from new sex‐linked genes. Genetics, 175, 1945–1954. 17287532 10.1534/genetics.106.070110 PMC1855140

[jeb14063-bib-0010] Bergero, R. , Qiu, S. , & Charlesworth, D. (2015). Gene loss from a plant sex chromosome system. Current Biology, 25, 1234–1240. 25913399 10.1016/j.cub.2015.03.015

[jeb14063-bib-0011] Bergero, R. , Qiu, S. , Forrest, A. , Borthwick, H. , & Charlesworth, D. (2013). Expansion of the pseudo‐autosomal region and ongoing recombination suppression in the *Silene latifolia* sex chromosomes. Genetics, 194, 673–686. 23733786 10.1534/genetics.113.150755 PMC3697972

[jeb14063-bib-0012] Bernasconi, G. , Antonovics, J. , Biere, A. , Charlesworth, D. , Delph, L. F. , Filatov, D. , Giraud, T. , Hood, M. E. , Marais, G. A. , McCauley, D. , Pannell, J. R. , Shykoff, J. A. , Vyskot, B. , Wolfe, L. M. , & Widmer, A. (2009). *Silene* as a model system in ecology and evolution. Heredity, 103, 5–14. 19367316 10.1038/hdy.2009.34

[jeb14063-bib-0013] Bolger, A. M. , Lohse, M. , & Usadel, B. (2014). trimmomatic: A flexible trimmer for Illumina sequence data. Bioinformatics, 30, 2114–2120. 24695404 10.1093/bioinformatics/btu170 PMC4103590

[jeb14063-bib-0014] Campos, J. L. , Qiu, S. , Guirao‐Rico, S. , Bergero, R. , & Charlesworth, D. (2017). Recombination changes at the boundaries of fully and partially sex‐linked regions between closely related *Silene* species pairs. Heredity, 118, 395–403. 27827389 10.1038/hdy.2016.113 PMC5345606

[jeb14063-bib-0015] Charlesworth, B. , Jordan, C. Y. , & Charlesworth, D. (2014). The evolutionary dynamics of sexually antagonistic mutations in pseudoautosomal regions of sex chromosomes. Evolution, 68, 1339–1350. 24476564 10.1111/evo.12364 PMC4289941

[jeb14063-bib-0016] Charlesworth, B. , & Wall, J. D. (1999). Inbreeding, heterozygote advantage and the evolution of neo‐X and neo‐Y sex chromosomes. Proceedings of the Royal Society B: Biological Sciences, 266, 51–56.

[jeb14063-bib-0017] Charlesworth, D. (2008). Sex chromosome origins and evolution. In M. Pagel & A. Pomiankowski (Eds.), Evolutionary genomics and proteomics (pp. 207–240). Sinauer Associates.

[jeb14063-bib-0018] Charlesworth, D. (2015). Plant contributions to our understanding of sex chromosome evolution. New Phytologist, 208, 52–65. 26053356 10.1111/nph.13497

[jeb14063-bib-0019] Charlesworth, D. (2017). Evolution of recombination rates between sex chromosomes. Philosophical Transactions of the Royal Society B, 372, 20160456. 10.1098/rstb.2016.0456 PMC5698619 29109220

[jeb14063-bib-0020] Charlesworth, D. (2018). The guppy sex chromosome system and the sexually antagonistic polymorphism hypothesis for Y chromosome recombination suppression. Genes, 9, 264. 29783761 10.3390/genes9050264 PMC5977204

[jeb14063-bib-0021] Charlesworth, D. (2021). When and how do sex‐linked regions become sex chromosomes? Evolution, 75, 569–581. 33592115 10.1111/evo.14196

[jeb14063-bib-0022] Chibalina, M. V. , & Filatov, D. A. (2011). Plant Y chromosome degeneration is retarded by haploid purifying selection. Current Biology, 21, 1475–1479. 21889890 10.1016/j.cub.2011.07.045

[jeb14063-bib-0023] Darolti, I. , Wright, A. E. , Sandkam, B. A. , Morris, J. , Bloch, N. I. , Farre, M. , Fuller, R. C. , Bourne, G. R. , Larkin, D. M. , Breden, F. , & Mank, J. E. (2019). Extreme heterogeneity in sex chromosome differentiation and dosage compensation in livebearers. Proceedings of the National Academy of Sciences of the United States of America, 116, 19031–19036. 31484763 10.1073/pnas.1905298116 PMC6754558

[jeb14063-bib-0024] Desfeux, C. , Maurice, S. , Henry, J. P. , Lejeune, B. , & Gouyon, P. H. (1996). Evolution of reproductive systems in the genus *Silene* . Proceedings of the Royal Society of London. Series B, 263, 409–414. 18386410 10.1098/rspb.1996.0062

[jeb14063-bib-0025] Edgar, R. C. (2004). MUSCLE: A multiple sequence alignment method with reduced time and space complexity. BMC Bioinformatics, 5, 113. 15318951 10.1186/1471-2105-5-113 PMC517706

[jeb14063-bib-0026] El Taher, A. , Ronco, F. , Matschiner, M. , Salzburger, W. , & Bohne, A. (2021). Dynamics of sex chromosome evolution in a rapid radiation of cichlid fishes. Science Advances, 7, eabe8215. 34516923 10.1126/sciadv.abe8215 PMC8442896

[jeb14063-bib-0027] Ellis, N. , Taylor, A. , Bengtsson, B. O. , Kidd, J. , Rogers, J. , & Goodfellow, P. (1990). Population structure of the human pseudoautosomal boundary. Nature, 344, 663–665. 2325773 10.1038/344663a0

[jeb14063-bib-0028] Filatov, D. A. (2005). Evolutionary history of *Silene latifolia* sex chromosomes revealed by genetic mapping of four genes. Genetics, 170, 975–979. 15834147 10.1534/genetics.104.037069 PMC1450409

[jeb14063-bib-0029] Filatov, D. A. (2009). Processing and population genetic analysis of multigenic datasets with proseq3 software. Bioinformatics, 25, 3189–3190. 19797407 10.1093/bioinformatics/btp572 PMC2778335

[jeb14063-bib-0030] Filatov, D. A. , & Gerrard, D. T. (2003). High mutation rates in human and ape pseudoautosomal genes. Gene, 317, 67–77. 14604793 10.1016/s0378-1119(03)00697-8

[jeb14063-bib-0031] Filatov, D. A. , Howell, E. C. , Groutides, C. , & Armstrong, S. J. (2009). Recent spread of a retrotransposon in the *Silene latifolia* genome, apart from the Y chromosome. Genetics, 181, 811–817. 19064703 10.1534/genetics.108.099267 PMC2644968

[jeb14063-bib-0032] Furman, B. L. S. , Metzger, D. C. H. , Darolti, I. , Wright, A. E. , Sandkam, B. A. , Almeida, P. , Shu, J. J. , & Mank, J. E. (2020). Sex chromosome evolution: So many exceptions to the rules. Genome Biology and Evolution, 12, 750–763. 32315410 10.1093/gbe/evaa081 PMC7268786

[jeb14063-bib-0033] Guirao‐Rico, S. , Sanchez‐Gracia, A. , & Charlesworth, D. (2017). Sequence diversity patterns suggesting balancing selection in partially sex‐linked genes of the plant *Silene latifolia* are not generated by demographic history or gene flow. Molecular Ecology, 26, 1357–1370. 28035715 10.1111/mec.13969

[jeb14063-bib-0034] Harkess, A. , Huang, K. , van der Hulst, R. , Tissen, B. , Caplan, J. L. , Koppula, A. , Batish, M. , Meyers, B. C. , & Leebens‐Mack, J. (2020). Sex determination by two Y‐linked genes in garden asparagus. Plant Cell, 32, 1790–1796. 32220850 10.1105/tpc.19.00859 PMC7268802

[jeb14063-bib-0035] Harkess, A. , Zhou, J. S. , Xu, C. Y. , Bowers, J. E. , Van der Hulst, R. , Ayyampalayam, S. , Mercati, F. , Riccardi, P. , McKain, M. R. , Kakrana, A. , Tang, H. B. , Ray, J. , Groenendijk, J. , Arikit, S. , Mathioni, S. M. , Nakano, M. , Shan, H. Y. , Telgmann‐Rauber, A. , Kanno, A. , … Chen, G. Y. (2017). The asparagus genome sheds light on the origin and evolution of a young Y chromosome. Nature Communications, 8, 1279. 10.1038/s41467-017-01064-8 PMC5665984 29093472

[jeb14063-bib-0036] Howell, E. C. , Armstrong, S. J. , & Filatov, D. A. (2009). Evolution of neo‐sex chromosomes in *Silene diclinis* . Genetics, 182, 1109–1115. 19448269 10.1534/genetics.109.103580 PMC2728852

[jeb14063-bib-0037] Hu, X. S. , & Filatov, D. A. (2016). The large‐X effect in plants: Increased species divergence and reduced gene flow on the *Silene* X‐chromosome. Molecular Ecology, 25, 2609–2619. 26479725 10.1111/mec.13427

[jeb14063-bib-0038] Hudson, R. R. , Boos, D. D. , & Kaplan, N. L. (1992). A statistical test for detecting geographic subdivision. Molecular Biology and Evolution, 9, 138–151. 1552836 10.1093/oxfordjournals.molbev.a040703

[jeb14063-bib-0039] Hudson, R. R. , Kreitman, M. , & Aguade, M. (1987). A test of neutral molecular evolution based on nucleotide data. Genetics, 116, 153–159. 3110004 10.1093/genetics/116.1.153 PMC1203113

[jeb14063-bib-0040] Ironside, J. E. (2010). No amicable divorce? Challenging the notion that sexual antagonism drives sex chromosome evolution. Bioessays, 32, 718–726. 20658710 10.1002/bies.200900124

[jeb14063-bib-0041] Jay, P. , Tezenas, E. , Véber, A. , & Giraud, T. (2022). Modeling the stepwise extension of recombination suppression on sex chromosomes and other supergenes through deleterious mutation sheltering. BioRxiv. 10.1101/2021.05.17.444504 PMC9295944 35853091

[jeb14063-bib-0042] Jeffries, D. L. , Gerchen, J. F. , Scharmann, M. , & Pannell, J. R. (2021). A neutral model for the loss of recombination on sex chromosomes. Philosophical Transactions of the Royal Society of London B: Biological Science, 376, 20200096. 10.1098/rstb.2020.0096 PMC8273504 34247504

[jeb14063-bib-0043] Jeffries, D. L. , Lavanchy, G. , Sermier, R. , Sredl, M. J. , Miura, I. , Borzee, A. , Barrow, L. N. , Canestrelli, D. , Crochet, P. A. , Dufresnes, C. , Fu, J. , Ma, W. J. , Garcia, C. M. , Ghali, K. , Nicieza, A. G. , O'Donnell, R. P. , Rodrigues, N. , Romano, A. , Martinez‐Solano, I. , … Perrin, N. (2018). A rapid rate of sex‐chromosome turnover and non‐random transitions in true frogs. Nature Communications, 9, 4088. 10.1038/s41467-018-06517-2 PMC6173717 30291233

[jeb14063-bib-0044] Kelly, J. K. (1997). A test of neutrality based on interlocus associations. Genetics, 146, 1197–1206. 9215920 10.1093/genetics/146.3.1197 PMC1208047

[jeb14063-bib-0045] Kirkpatrick, M. , & Guerrero, R. F. (2014). Signatures of sex‐antagonistic selection on recombining sex chromosomes. Genetics, 197, 531–541. 24578352 10.1534/genetics.113.156026 PMC4063913

[jeb14063-bib-0046] Kirkpatrick, M. , Guerrero, R. F. , & Scarpino, S. V. (2010). Patterns of neutral genetic variation on recombining sex chromosomes. Genetics, 184, 1141–1152. 20124026 10.1534/genetics.109.113555 PMC2865914

[jeb14063-bib-0047] Krasovec, M. , Chester, M. , Ridout, K. , & Filatov, D. A. (2018a). The mutation rate and the age of the sex chromosomes in *Silene latifolia* . Current Biology, 28, 1832–1838. 29804812 10.1016/j.cub.2018.04.069

[jeb14063-bib-0048] Krasovec, M. , Nevado, B. , & Filatov, D. A. (2018b). A comparison of selective pressures in plant X‐linked and autosomal genes. Genes, 9, 234. 29751495 10.3390/genes9050234 PMC5977174

[jeb14063-bib-0049] Krasovec, M. , Zhang, Y. , & Filatov, D. A. (2020). The location of the pseudoautosomal boundary in *Silene latifolia* . Genes, 11, 610. 32486434 10.3390/genes11060610 PMC7348893

[jeb14063-bib-0050] Kratochvil, L. , Stock, M. , Rovatsos, M. , Bullejos, M. , Herpin, A. , Jeffries, D. L. , Peichel, C. L. , Perrin, N. , Valenzuela, N. , & Pokorna, M. J. (2021). Expanding the classical paradigm: What we have learnt from vertebrates about sex chromosome evolution. Philosophical Transactions of the Royal Society of London B: Biological Science, 376, 20200097. 10.1098/rstb.2020.0097 PMC8310716 34304593

[jeb14063-bib-0051] Kumar, S. , Stecher, G. , Li, M. , Knyaz, C. , & Tamura, K. (2018). mega X: Molecular evolutionary genetics analysis across computing platforms. Molecular Biology and Evolution, 35, 1547–1549. 29722887 10.1093/molbev/msy096 PMC5967553

[jeb14063-bib-0052] Langmead, B. , & Salzberg, S. L. (2012). Fast gapped‐read alignment with Bowtie 2. Nature Methods, 9, 357–359. 22388286 10.1038/nmeth.1923 PMC3322381

[jeb14063-bib-0053] Lahn, B. T. , & Page, D. C. (1999). Four evolutionary strata on the human X chromosome. Science, 286, 964–967. 10542153 10.1126/science.286.5441.964

[jeb14063-bib-0054] Lappin, F. M. , Medert, C. M. , Hawkins, K. K. , Mardonovich, S. , Wu, M. , & Moore, R. C. (2015). A polymorphic pseudoautosomal boundary in the *Carica papaya* sex chromosomes. Molecular Genetics and Genomics, 290, 1511–1522. 25711306 10.1007/s00438-015-1000-3

[jeb14063-bib-0055] Lenormand, T. , & Roze, D. (2022). Y recombination arrest and degeneration in the absence of sexual dimorphism. Science, 375, 663–666. 35143289 10.1126/science.abj1813

[jeb14063-bib-0056] Li, B. , & Dewey, C. N. (2011). RSEM: Accurate transcript quantification from RNA‐Seq data with or without a reference genome. BMC Bioinformatics, 12, 323. 21816040 10.1186/1471-2105-12-323 PMC3163565

[jeb14063-bib-0057] Lien, S. , Szyda, J. , Schechinger, B. , Rappold, G. , & Arnheim, N. (2000). Evidence for heterogeneity in recombination in the human pseudoautosomal region: High resolution analysis by sperm typing and radiation‐hybrid mapping. American Journal of Human Genetics, 66, 557–566. 10677316 10.1086/302754 PMC1288109

[jeb14063-bib-0058] Liu, X. , Glemin, S. , & Karrenberg, S. (2020). Evolution of putative barrier loci at an intermediate stage of speciation with gene flow in campions (*Silene*). Molecular Ecology, 29, 3511–3525. 32740990 10.1111/mec.15571 PMC7540528

[jeb14063-bib-0059] Luthringer, R. , Lipinska, A. P. , Roze, D. , Cormier, A. , Macaisne, N. , Peters, A. F. , Cock, J. M. , & Coelho, S. M. (2015). The pseudoautosomal regions of the U/V sex chromosomes of the brown alga *Ectocarpus* exhibit unusual features. Molecular Biology and Evolution, 32, 2973–2985. 26248564 10.1093/molbev/msv173 PMC4610043

[jeb14063-bib-0060] Mank, J. E. , Hosken, D. J. , & Wedell, N. (2014). Conflict on the sex chromosomes: Cause, effect, and complexity. Cold Spring Harbor Perspectives in Biology, 6, a017715. 25280765 10.1101/cshperspect.a017715 PMC4292157

[jeb14063-bib-0061] McKenna, A. , Hanna, M. , Banks, E. , Sivachenko, A. , Cibulskis, K. , Kernytsky, A. , Garimella, K. , Altshuler, D. , Gabriel, S. , Daly, M. , & DePristo, M. A. (2010). The Genome Analysis Toolkit: a MapReduce framework for analyzing next‐generation DNA sequencing data. Genome Res, 20, 1297–303. 20644199 10.1101/gr.107524.110 PMC2928508

[jeb14063-bib-0062] Monteiro, B. , Arenas, M. , Prata, M. J. , & Amorim, A. (2021). Evolutionary dynamics of the human pseudoautosomal regions. PLoS Genetics, 17, e1009532. 33872316 10.1371/journal.pgen.1009532 PMC8084340

[jeb14063-bib-0063] Morgan, A. P. , Bell, T. A. , Crowley, J. J. , & Pardo‐Manuel de Villena, F. (2019). Instability of the pseudoautosomal boundary in house mice. Genetics, 212, 469–487. 31028113 10.1534/genetics.119.302232 PMC6553833

[jeb14063-bib-0064] Muir, G. , Dixon, C. J. , Harper, A. L. , & Filatov, D. A. (2012). Dynamics of drift, gene flow, and selection during speciation in *Silene* . Evolution, 66, 1447–1458. 22519783 10.1111/j.1558-5646.2011.01529.x

[jeb14063-bib-0065] Muller, N. A. , Kersten, B. , Leite Montalvao, A. P. , Mahler, N. , Bernhardsson, C. , Brautigam, K. , Carracedo Lorenzo, Z. , Hoenicka, H. , Kumar, V. , Mader, M. , Pakull, B. , Robinson, K. M. , Sabatti, M. , Vettori, C. , Ingvarsson, P. K. , Cronk, Q. , Street, N. R. , & Fladung, M. (2020). A single gene underlies the dynamic evolution of poplar sex determination. Nature Plants, 6, 630–637. 32483326 10.1038/s41477-020-0672-9

[jeb14063-bib-0066] Nei, M. (1987). Molecular evolutionary genetics. Columbia University Press.

[jeb14063-bib-0067] Ohno, S. (1967). Sex chromosomes and sex‐linked genes. Springer‐Verlag.

[jeb14063-bib-0068] Olito, C. , & Abbott, J. K. (2020). The evolution of suppressed recombination between sex chromosomes by chromosomal inversions. bioRxiv. 10.1101/2020.03.23.003558 36794986

[jeb14063-bib-0069] Olito, C. , Ponnikas, S. , Hansson, B. , & Abbott, J. K. (2022). Consequences of recessive deleterious genetic variation for the evolution of inversions suppressing recombination between sex chromosomes. Evolution, 76, 1320–1330. 10.1111/evo.14496 35482933 PMC9324078

[jeb14063-bib-0070] Otto, S. P. , Pannell, J. R. , Peichel, C. L. , Ashman, T. L. , Charlesworth, D. , Chippindale, A. K. , Delph, L. F. , Guerrero, R. F. , Scarpino, S. V. , & McAllister, B. F. (2011). About PAR: The distinct evolutionary dynamics of the pseudoautosomal region. Trends in Genetics, 27, 358–367. 21962971 10.1016/j.tig.2011.05.001

[jeb14063-bib-0071] Papadopulos, A. S. , Chester, M. , Ridout, K. , & Filatov, D. A. (2015). Rapid Y degeneration and dosage compensation in plant sex chromosomes. Proceedings of the National Academy of Sciences of the United States of America, 112, 13021–13026. 26438872 10.1073/pnas.1508454112 PMC4620866

[jeb14063-bib-0072] Ponnikas, S. , Sigeman, H. , Abbott, J. K. , & Hansson, B. (2018). Why do sex chromosomes stop recombining? Trends in Genetics, 34, 492–503. 29716744 10.1016/j.tig.2018.04.001

[jeb14063-bib-0073] Qiu, S. , Bergero, R. , & Charlesworth, D. (2013). Testing for the footprint of sexually antagonistic polymorphisms in the pseudoautosomal region of a plant sex chromosome pair. Genetics, 194, 663–672. 23733787 10.1534/genetics.113.152397 PMC3697971

[jeb14063-bib-0074] Qiu, S. , Bergero, R. , Guirao‐Rico, S. , Campos, J. L. , Cezard, T. , Gharbi, K. , & Charlesworth, D. (2016). RAD mapping reveals an evolving, polymorphic and fuzzy boundary of a plant pseudoautosomal region. Molecular Ecology, 25, 414–430. 26139514 10.1111/mec.13297

[jeb14063-bib-0075] Rice, W. R. (1987). The accumulation of sexually antagonistic genes as a selective agent promoting the evolution of reduced recombination between primitive sex chromosomes. Evolution, 41, 911–914. 28564364 10.1111/j.1558-5646.1987.tb05864.x

[jeb14063-bib-0076] Rodrigues, N. , Studer, T. , Dufresnes, C. , & Perrin, N. (2017). Sex‐chromosome recombination in common frogs brings water to the fountain‐of‐youth. Molecular Biology and Evolution, 35, 942–948. 10.1093/molbev/msy008 29394416

[jeb14063-bib-0077] Rozas, J. , Ferrer‐Mata, A. , Sanchez‐DelBarrio, J. C. , Guirao‐Rico, S. , Librado, P. , Ramos‐Onsins, S. E. , & Sanchez‐Gracia, A. (2017). dnasp 6: DNA sequence polymorphism analysis of large data sets. Molecular Biology and Evolution, 34, 3299–3302. 29029172 10.1093/molbev/msx248

[jeb14063-bib-0078] Sierro, N. , Battey, J. N. , Ouadi, S. , Bakaher, N. , Bovet, L. , Willig, A. , Goepfert, S. , Peitsch, M. C. , & Ivanov, N. V. (2014). The tobacco genome sequence and its comparison with those of tomato and potato. Nature Communications, 5, 3833. 10.1038/ncomms4833 PMC4024737 24807620

[jeb14063-bib-0079] Tajima, F. (1989). Statistical method for testing the neutral mutation hypothesis by DNA polymorphism. Genetics, 123, 585–595. 2513255 10.1093/genetics/123.3.585 PMC1203831

[jeb14063-bib-0080] Vicoso, B. (2019). Molecular and evolutionary dynamics of animal sex‐chromosome turnover. Nature Ecology and Evolution, 3, 1632–1641. 31768022 10.1038/s41559-019-1050-8

[jeb14063-bib-0081] Wakeley, J. (1998). Segregating sites in Wright's island model. Theoretical Population Biology, 53, 166–174. 9615475 10.1006/tpbi.1997.1355

[jeb14063-bib-0082] Warmke, H. E. (1946). Sex determination and sex balance in *Melandrium* . American Journal of Botany, 33, 648–660.

[jeb14063-bib-0083] Westergaard, M. (1946). Aberrant Y chromosomes and sex expression in *Melandrium album* . Hereditas, 32, 419–443. 20998142 10.1111/j.1601-5223.1946.tb02784.x

[jeb14063-bib-0084] Westergaard, M. (1958). The mechanism of sex determination in dioecious flowering plants. Advanced Genetics, 9, 217–281. 10.1016/s0065-2660(08)60163-7 13520443

[jeb14063-bib-0085] Wright, A. E. , Dean, R. , Zimmer, F. , & Mank, J. E. (2016). How to make a sex chromosome. Nature Communications, 7, 12087. 10.1038/ncomms12087 PMC4932193 27373494

[jeb14063-bib-0086] Wright, S. I. , & Charlesworth, B. (2004). The HKA test revisited: A maximum‐likelihood‐ratio test of the standard neutral model. Genetics, 168, 1071–1076. 15514076 10.1534/genetics.104.026500 PMC1448833

[jeb14063-bib-0087] Zhou, Q. , Zhang, J. , Bachtrog, D. , An, N. , Huang, Q. , Jarvis, E. D. , Gilbert, M. T. P. , & Zhang, G. (2014). Complex evolutionary trajectories of sex chromosomes across bird taxa. Science, 346, 1332. 10.1126/science.1246338 PMC6445272 25504727

