# Supplementary material for: Recent expansion of the non‐recombining sex‐linked region on Silene latifolia sex chromosomes
Source: J Evol Biol. 2022 Jul 14;35(12):1696–708. doi: 10.1111/jeb.14063 (PMC10083954; doi:10.1111/jeb.14063)
Supplement: Supplementary file 1 — Figure S1 [file JEB-35-1696-s002.pdf]

Supplementary figures and tables

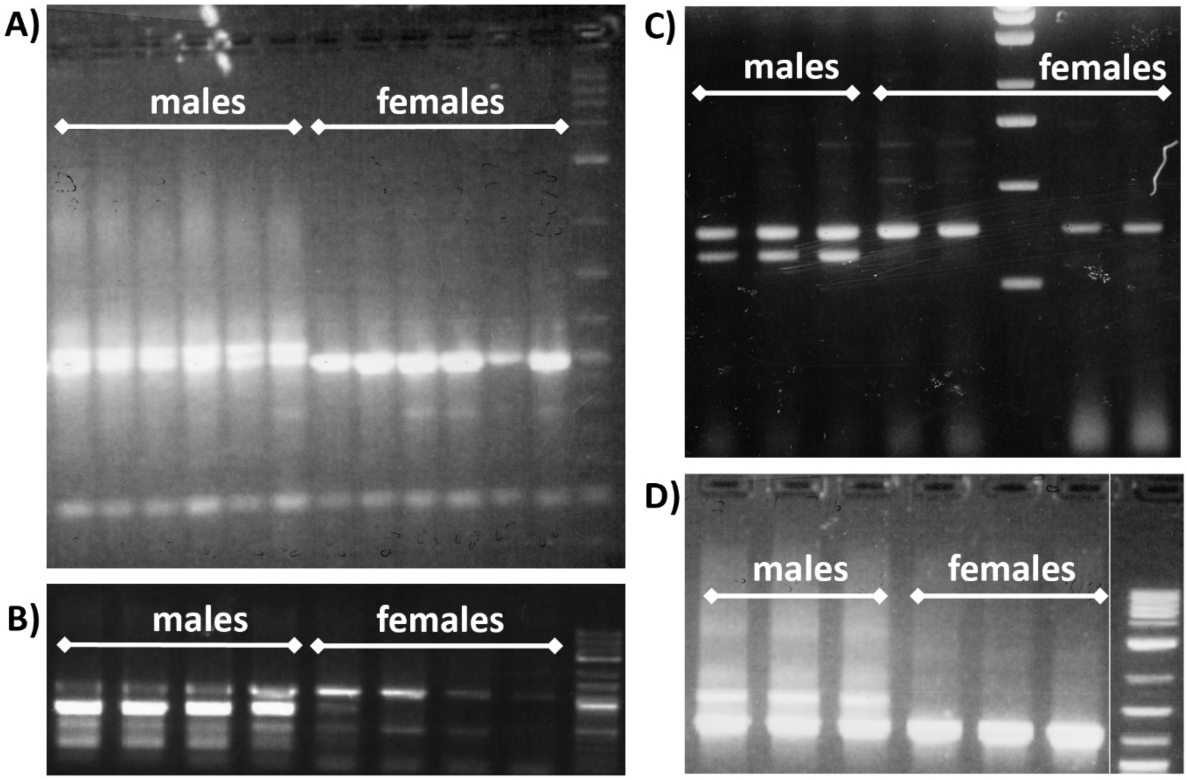

Figure S1. Experimental verification of male-specificity of Y-linked genes A) contig1046Y, B) contig9011Y, C) contig12495Y and D) contig528Y in randomly chosen unrelated male and female *S. latifolia* plants.

Supplementary tables S1-S4 (provided as a single excel file).

Table S1. Transcriptome sequence data used in the analyses.

Table S2. Synonymous DNA sequence polymorphism in 57 genes near the PAR boundary in *S. latifolia* and *S. dioica*.

Table S3. Patterns of DNA sequence polymorphism in 57 genes near the PAR boundary in *S. latifolia* and *S. dioica*.

Table S4. DNA sequence polymorphism in 30 autosomal genes mapped by Papadopulos et al (2016), in *S. latifolia* and *S. dioica.*
